# Metagenome-Assembled Genomes of Bacteria Associated with Massospora cicadina Fungal Plugs from Infected Brood VIII Periodical Cicadas

**DOI:** 10.1128/mra.00413-22

**Published:** 2022-08-29

**Authors:** Cassandra L. Ettinger, Brian Lovett, Matt T. Kasson, Jason E. Stajich

**Affiliations:** a Department of Microbiology and Plant Pathology, University of California—Riverside, Riverside, California, USA; b Institute for Integrative Genome Biology, University of California—Riverside, Riverside, California, USA; c Division of Plant and Soil Sciences, West Virginia University, Morgantown, West Virginia, USA; Vanderbilt University

## Abstract

We report six metagenome-assembled genomes (MAGs) associated with Massospora cicadina strain MCPNR19 (ARSEF 14555), an obligate entomopathogenic fungus of periodical cicadas. The MAGs include representatives of *Pantoea*, Pseudomonas, *Lactococcus*, and one potential new *Chryseobacterium* species. Future research is needed to resolve the ecology of these MAGs and determine whether they represent symbionts or contaminants.

## ANNOUNCEMENT

Massospora cicadina (Zoopagomycota) is an understudied obligate entomopathogenic fungus that infects periodical cicadas (1, 2). During assembly and annotation of an improved genomic resource for *M. cicadina* strain MCPNR19 (ARSEF 14555) (3) using BlobTools2 (4), we identified many bacterial contigs. Given the discovery of psychoactive compounds present in *Massospora* species (5) and the uncertainty regarding their production, coupled with the established relationships between cicadas and bacterial mutualists (6), we sought to bin metagenome-assembled genomes (MAGs) from the improved *M. cicadina* genome to inform future investigations into tripartite cicada-*Massospora*-bacteria interactions.

The sampling, extraction, sequencing, quality control, and assembly methods are described by Stajich et al. (3). Briefly, conidia and azygospores of *M. cicadina* MCPNR19 (ARSEF 14555) were collected from fungal plugs of infected brood XIII Pharaoh cicadas (Magicicada septendecim) in June 2019. Genomic DNA from spores was sequenced using both Illumina and Nanopore technologies. The Nanopore data were assembled using wtdbg2 v. 2.5 (7), followed by multiple rounds of polishing with the Illumina reads (8).

We screened the preliminary *M. cicadina* genome assembly from Stajich et al. (3) for MAGs using the Anvi’o v. 7 pipeline (9). First, we calculated the genomic coverage against the Illumina reads using Bowtie2 v. 2.4.2 (10) and SAMtools v. 1.11 (11). We then used “anvi-gen-contigs-database” to generate a database from the *M. cicadina* genome assembly and called open-reading frames on this database using Prodigal v. 2.6.3 (12). As part of the Anvi’o pipeline, we identified bacterial (13), archaeal (13), and protista (14) single-copy genes using HMMER v. 3.2.1 (15) and rRNA genes using Barrnap (16). A predicted taxonomy was assigned to each gene call using Kaiju v. 1.7.2 (17) with the NCBI BLAST nonredundant (nr) protein database v. 2020-05-25, which included fungi and microbial eukaryotes. An Anvi’o profile was then constructed using “anvi-profile” for contigs >2.5 kbp with the “–cluster-contigs” option. The automatic binning algorithm, MetaBAT2 v. 2.12.1 (18), was run on contigs >2.5 kbp from the *M. cicadina* genome assembly to generate the preliminary MAGs. These MAGs were imported into Anvi’o using “anvi-import-collection” and were then manually inspected, combined, and refined using “anvi-interactive” and “anvi-refine.” The completeness and redundancy of the MAGs was assessed within Anvi’o using “anvi-summarize” and then again using the CheckM v. 1.1.3 lineage-specific workflow (19). We defined the MAGs as high quality if they were >90% complete, medium quality if >50% complete, and low quality if <50% complete as in reference 20. To obtain a putative taxonomy for each MAG, we used GTDB-Tk v. 1.5.0 (21), which places MAGs phylogenetically in the context of the Genome Taxonomy Database. We visualized the MAG phylogenetic placement in R v. 4.1.2 using the ggtree v. 3.2.1 and Treeio v. 1.18.1 packages (22–24).

**FIG 1:**
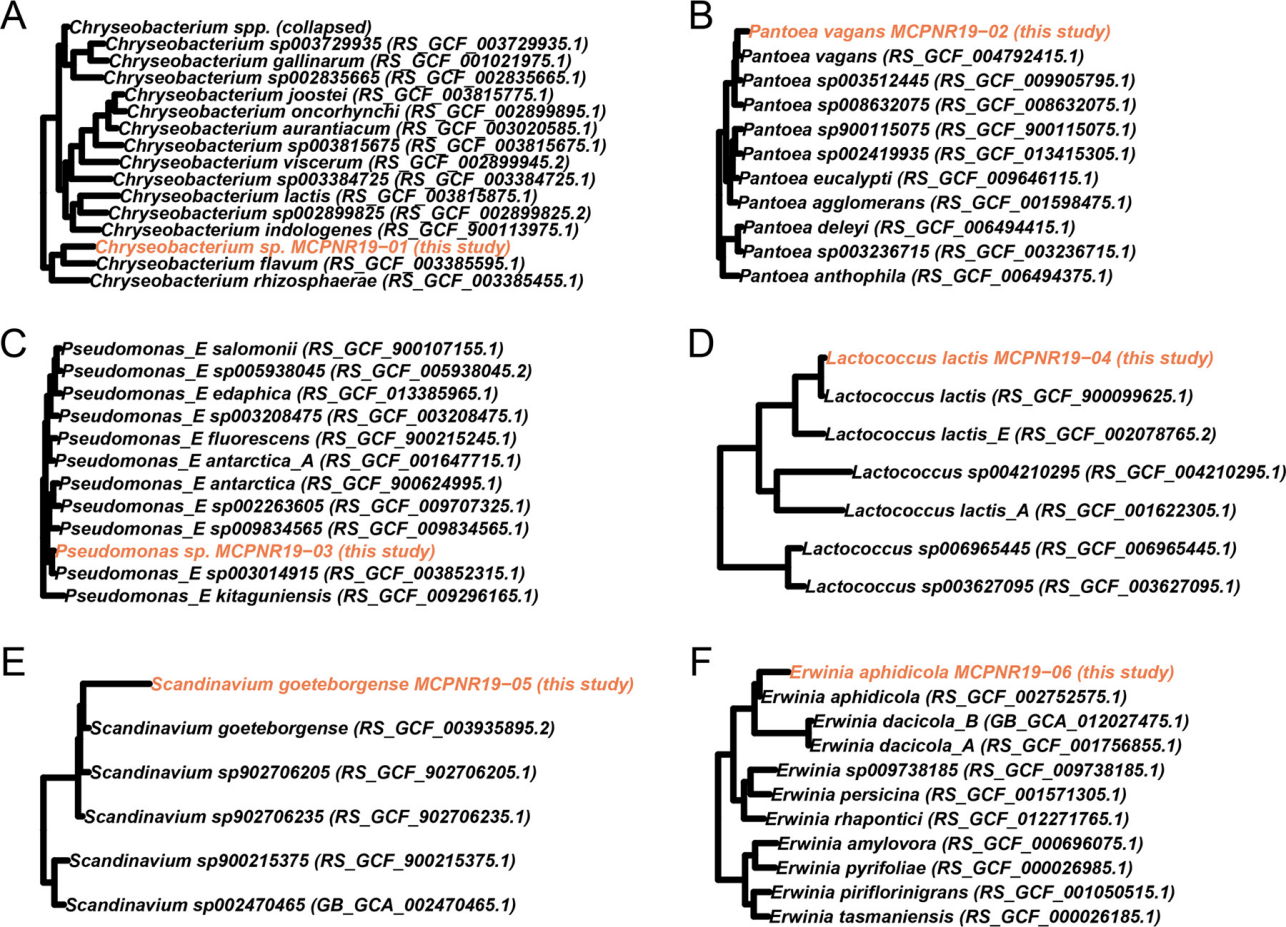
Phylogenetic placement of draft MAGs using GTDB-Tk. Phylogenetic trees were produced using GTDB-Tk for (A) *Chryseobacterium* sp. MCPNR19-01, (B) Pantoea vagans MCPNR19-02, (C) Pseudomonas sp. strain MCPNR19-03, (D) Lactococcus lactis MCPNR19-04, (E) Scandinavium goeteborgense MCPNR19-05, and (F) Erwinia aphidicola MCPNR19-06. MAGs generated in this study are highlighted in orange.

We generated a total of six draft MAGs (two high-quality and single-contig, two medium-quality, and two low-quality MAGs) representing six genera, including a high-quality MAG for a potentially new *Chryseobacterium* species (Fig. 1; Table 1). No MAGs were obtained representing the known cicada bacterial mutualists *Hodgkinia* or *Sulcia*. The higher genomic coverage of MAGs in the azygospore sequencing libraries may indicate that these MAGs represent opportunistic infections of moribund cicadas. Ultimately, these MAGs will provide valuable future insight into *Massospora*-bacterial interactions and symbiosis.

**TABLE 1:** Summary of genomic features for metagenome-assembled genomes identified in association with Massospora cicadina

Bin identifier	Draft quality^*a*^	Putative taxonomy	Closest taxonomic placement ANI (%)^*b*^	Genome size (bp)	No. of contigs	*N*_50_ (bp)	GC content (%)	Illumina mean coverage (×)^*c*^	Nanopore mean coverage (×)^*d*^		No. of genes^*e*^	CheckM % completion	CheckM % redundancy	Anvi’o % completion	Anvi’o % redundancy	GenBank accession no. or reference
									Azygospores	Conidia						
MCPNR19-01	High	*Chryseobacterium* sp.	82.96	4,875,107	1	4,875,107	37.60	16.11	25.62	2.46	4,647	100	0.61	97.18	2.82	JALIHL010000000
MCPNR19-02	High	Pantoea vagans	97.36	3,966,629	1	3,966,629	55.49	43.32	56.22	0.89	3,824	93.42	0.08	98.59	2.82	JALIHM010000000
MCPNR19-03	Medium	Pseudomonas sp.	95.96	6,113,041	63	666,019	60.52	316.80	102.70	0.03	5,730	87.25	2.49	95.78	0	JALIHN010000000
MCPNR19-04	Medium	Lactococcus lactis	98.38	2,214,970	5	892,863	34.95	5.22	28.33	0.99	2,560	85.29	1.7	94.37	2.82	JALIHO010000000
MCPNR19-05	Low	Scandinavium goeteborgense	95.47	1,822,081	68	39,073	55.75	21.35	19.65	1.42	2,118	45.61	0	35.21	0	25
MCPNR19-06	Low	Erwinia aphidicola	97.49	1,786,331	62	35,544	56.90	22.98	15.83	6.16	1,991	29.31	0	30.99	1.41	25

a Draft quality was assigned based on CheckM completion and redundancy estimates.

b Average nucleotide identity (ANI) to closest taxonomic placement in the Genome Taxonomy Database as reported by GTDB-Tk.

c Illumina data sets were generated from fungal azygospores.

d Nanopore data sets were generated from either fungal azyogospores or conidia.

e Number of genes predicted using Prodigal.

### Data availability.

The sequence reads have been deposited under SRA accession numbers SRR17553520 to SRR17553526 and BioProject accession number PRJNA795459. The four medium- and high-quality MAG assemblies have been deposited at DDBJ/ENA/GenBank under accession numbers JALIHL000000000, JALIHM000000000, JALIHN000000000, and JALIHO000000000. The versions described in this paper are JALIHL010000000, JALIHM010000000, JALIHN010000000, and JALIHO010000000. The two low-quality MAG assemblies are archived at Zenodo (25). Related computational scripts for this work are available on GitHub and archived in Zenodo (26).
